# Three-Dimensional Slope Imaging Method for Ground-Based Real-Aperture Radar

**DOI:** 10.3390/s21103511

**Published:** 2021-05-18

**Authors:** Hao Zhang, Xiaolin Yang, Feng Yang, Haitao Ma, Zhengxing Yu, Xiangtian Zheng, Yuan Sun

**Affiliations:** 1 School of Mechanical Electronic and Information Engineering, China University of Mining and Technology (Beijing), Beijing 100083, China; zhstrive@student.cumtb.edu.cn (H.Z.); yangf@cumtb.edu.cn (F.Y.); tbp150404024@student.cumtb.edu.cn (Y.S.); 2 China Academy of Safety Science and Technology, Beijing 100012, China; maht@chinasafety.ac.cn (H.M.); yuzx@chinasafety.ac.cn (Z.Y.); 3 School of Earth Science and Engineering, Hohai University, Nanjing 211100, China; zhengxt@hhu.edu.cn

**Keywords:** slope monitoring, ground-based real-aperture radar, smoothness constraint, three-dimensional imaging

## Abstract

Traditional two-dimensional radar images can only reflect the target azimuth and slant range and thus suffer problems of geometric deformation and overlapping. The unique three-dimensional (3D) imaging capability of ground-based real-aperture radar can more accurately and directly achieve correlation between the radar image and the slope monitoring scenarios, thus providing reliable information for the early warning and forecasting of landslides and collapse disasters. The latest method of selecting a slope target from a high-resolution range profile includes two indexes: maximum amplitude and coherence, which will affect the accuracy of displacement measurement when there is an interference target. We present a three-dimensional slope imaging method based on smoothness constraints. On the basis of the latest method, the objective fact of the practically smooth and continuous distribution of slope surfaces is considered. This method can be used for image interpretation on strongly scattered targets within the slope. The independently developed ground-based real-aperture slope radar system was deployed in the Heidaigou Open-Pit Coal Mine in Inner Mongolia to carry out 3D slope imaging experiments. The effectiveness of this method in slope monitoring and imaging was confirmed by comparing the surface roughness and the spatial positions of the targets with the high-density point cloud data in the projective plane obtained during the same time period. We used RMSE function and roughness as two measures. It shows that the method presented in this paper is more suitable for actual three-dimensional slope imaging.

## 1. Introduction

As a result of rapid economic development, along with the impact of natural factors, landslide disasters in China have been increasing year by year [1,2,3]. The three-dimensional (3D) imaging of slopes enables monitoring personnel to judge the slope deformation and displacement visually. It also improves the efficacy of stability analysis conducted on hidden slopes and is extremely important for developing landslide disaster early warning and prediction systems.

Microwave remote sensing technology is widely used in various fields involving slope deformation monitoring due to its high precision, all-weather characteristics, and other theoretical advantages. However, conventional radar can only acquire two-dimensional (2D) projection images of targets in the slope space on the slant range–azimuth plane. The use of 3D laser scanners, drones, and other topographic mapping methods is required to overcome image issues, such as overlapping, foreshortening, and shadows, caused by the lack of elevation information of the scattering target. These image issues also cause problems in engineering applications and emergency rescue work [4,5,6,7,8,9].

**Figure 1:**
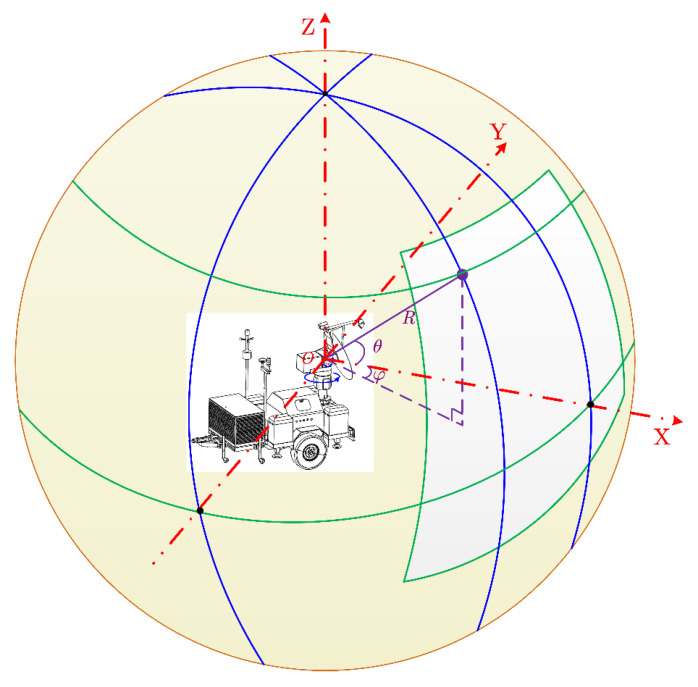
Imaging geometry model of the ground-based real-aperture radar system.

As shown in Figure 1, ground-based real-aperture radar can quickly obtain the spatial distribution of strongly scattered targets in the slope region using precise coordination between a radio frequency (RF) transceiver system and a servo turntable system. This process is beneficial for omnidirectional 3D recognition and imaging and has the advantage of strong mobility, and is especially suitable for the deformation monitoring of large-area, short-distance high, and steep slopes (such as the slopes of open-pit mines and tailing ponds) generated by modern geotechnical engineering.

Australia, South Africa, and other countries all have mature ground-based real-aperture radar systems [10,11,12,13,14,15,16], but there is no publicly available literature on relevant radar 3D imaging processing methods or evaluations of imaging results. The latest method of selecting a slope target from a high-resolution range profile includes two indexes: maximum amplitude and coherence, which will affect the accuracy of displacement measurement when there is an interference target.

This paper presents a 3D imaging method based on slope smoothness constraints. A ground-based real-aperture slope radar system prototype, S-RAR, which was designed and developed by the Chinese Academy of Safety Sciences and Technology, was used in this research. The remainder of the paper is arranged as follows: Section 2 briefly describes the radar echo signal model and the content related to time-frequency processing. The third section summarizes the 3D imaging process based on an analysis of the slope smoothness constraint principle, and real slope data processing results are displayed for each major step.

The fourth section compares and analyzes the 3D imaging results of the high and steep slopes of the Heidaigou Open-Pit Coal Mine in Inner Mongolia with the 3D point cloud data acquired during the same period. The results show that the spatial positions of the radar imaging targets and the strongly scattered targets of the slope basically correspond in an accurate fashion, thus verifying the 3D imaging capability of the radar system and the effectiveness of the proposed method. The fifth section summarizes the content of the article.

## 2. Background Knowledge

Ground-based real-aperture radar continuously monitors slopes using a wide-range point source scanning method. $A\left(\theta_{m},\varphi_{n}\right)$ denotes the weight of the target echo amplitude and $R\left(\theta_{m},\varphi_{n}\right)$ indicates the distance from the target to the line of sight of the radar. Under elevation angle θ and azimuth angle φ, the received scattered echo signal model can be expressed as follows:

(1) $$S\left(f_{k},\theta_{m},\varphi_{n}\right)=A\left(\theta_{m},\varphi_{n}\right)\cdot \exp\left\{-j4\pi f_{k}R\left(\theta_{m},\varphi_{n}\right)/c\right\}$$

where $f_{k}$ is the frequency of the scattered echo signal, k=1,2,…K; $\theta_{m}$ is the elevation angle of the radar, m=1,2,…M; $\varphi_{n}$ is the horizontal angle of the radar, n=1,2,…N; exp{⋅} represents the natural exponential function; j represents the imaginary unit; and c represents the electromagnetic wave velocity in free space.

The ground-based real-aperture radar system transmits a radar signal at a frequency increment of Δf within the set frequency bandwidth. The range resolution Δr between frequency bandwidth B and the radar can be expressed as follows:

(2) $$\Delta r=\frac{v}{2B}$$

where v represents the speed of the electromagnetic wave signal in the propagation medium ($v\approx c=3\times 10^{8}$ m/s in the air).

### 2.1. Frequency Domain Windowing

Windowing is also called weighting. The essence of this process is that the real-valued function moves symmetrically from the apex of the signal center to both ends. The weighting of the center is greater than the weighting of the end of the window to alleviate the spectral energy leakage caused by the sudden truncation of the finite length sequence in the signal processing, thus reducing the target sidelobe amplitude [17].

The Kaiser window has parameters for adjusting the degree of weighting, which can balance the needs of reducing the sidelobes and widening the resolution. A Kaiser window of length *T* applied in the time domain can be defined as follows [18]:

(3) $$w_{k}\left(t,T\right)=\frac{I_{o}\left(\beta \sqrt{1-{\left(2t/T\right)}^{2}}\right)}{I_{o}\left(\beta \right)},t\in \left[-\frac{T}{2},\frac{T}{2}\right]$$

In the formula, β is the adjustable sliding coefficient or smoothing coefficient, and $I_{o}\left(\cdot \right)$ is the zero-order Bessel function. Similarly, the Kaiser window of length *F* applied in the frequency domain can be defined as follows:

(4) $$W_{k}\left(f,F\right)=\frac{I_{o}\left(\beta \sqrt{1-{\left(2f/F\right)}^{2}}\right)}{I_{o}\left(\beta \right)},f\in \left[-\frac{F}{2},\frac{F}{2}\right]$$

Frequency-domain Kaiser windowing of the radar scattered echo can be expressed as follows:

(5) $$S^{\prime }\left(f_{k},\theta_{m},\varphi_{n}\right)=S\left(f_{k},\theta_{m},\varphi_{n}\right)\cdot W\left(k,\beta \right)$$

where W(k,β), which is based on the smoothing coefficient β, can create a column vector containing k elements within MATLAB. When designing the performance of a system, the impulse response width (IRW) and peak sidelobe ratio have restrictions [19]. Therefore, trade-offs must be made when selecting the parameters of the window. Table 1 lists some of the Kaiser window parameters.

### 2.2. One-Dimensional Imaging Processing

At any time, the Fourier transform can complete the equivalent measurement of target scattering characteristics in the time and frequency domains [20].

The scattering echo signal of the radar spot is orthogonally demodulated and then subjected to Inverse Fast Fourier Transform (IFFT). The distribution of the target scattering center on each range unit, which is also referred to as the high-resolution range profile (HRRP), can be obtained as follows [21]:

(6) $$s\left(R_{\ell },\theta_{m},\varphi_{n}\right)=\mathrm{IFFT}\left\{S\left(f_{k},\theta_{m},\varphi_{n}\right)\right\}=\frac{1}{L}\overset{K}{\underset{k=1}{\sum }}S\left(f_{k},\theta_{m},\varphi_{n}\right)\exp\left\{j\frac{2\pi }{L}k\frac{R_{\ell }L}{c/2\Delta f}\right\}=\frac{A}{L}\cdot \exp\left\{-j\frac{4\pi f_{c}}{c}R\left(\theta_{m},\varphi_{n}\right)\right\}\cdot \frac{\sin\left(\pi ka\right)}{\sin\left(\pi a\right)}$$

where, $f_{c}=f_{1}+\left(K-1\right)\Delta f/2$ represents the average frequency, which can be regarded as the carrier frequency of the echo signal; $\exp\left\{-j4\pi f_{c}R\left(\theta_{m},\varphi_{n}\right)/c\right\}$ is the fixed phase factor; and $a=2\Delta f\left(R\left(\theta_{m},\varphi_{n}\right)-R_{\ell }\right)/c$, when a=0, $R_{\ell }=R\left(\theta_{m},\varphi_{n}\right)$. At this point, the electrical length ΔR of the RF chain within the radar system is subtracted to obtain the distance between the origin of the radar coordinate and the target’s main scattering center.

**Figure 2:**
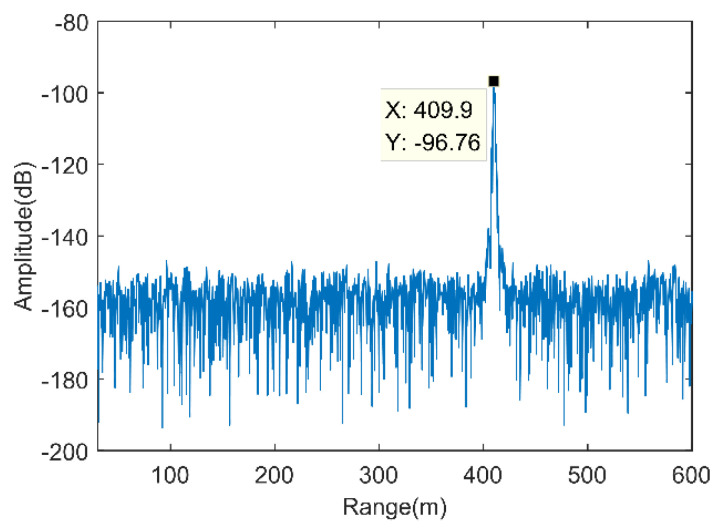
High-resolution range profile.

The HRRP of the ground-based real-aperture radar monitoring of the high-step slope of the Heidaigou Open-Pit Coal Mine at the preset monitoring direction $\left(\theta_{m},\varphi_{n}\right)$ is shown in Figure 2. From the figure, the line of sight between the slope and the phase center of the radar antenna can be roughly distinguished. The directional distance is approximately 409.9 m, which is basically consistent with the results obtained by the laser rangefinder in the same period.

The range gate was a bandpass time filter, which can be used to observe a portion of the time-domain response while shielding the redundant interference portion [22]. The influence of other clutter outside the spot monitoring area on the extraction of the target at the time-domain peak point can be reduced by adding a gate in the time domain. The width of the range gate must include most of the energy of the target in the slope space. Therefore, selecting an appropriate time period according to actual conditions is the key issue for time-domain gating.

Within the preset monitoring range, the distance between the high-step slope of the Heidaigou Open-Pit Coal Mine and the line of sight of the S-RAR radar pan–tilt platform was approximately 300–540 m, as measured by a laser rangefinder.

### 2.3. Coherence Analysis

The amplitude and phase stability of scattering targets can be better reflected by using coherence. In this paper, coherence was used as an evaluation criterion to further screen the time-domain peak point set filtered by the normalized amplitude threshold. The coherence of radar images acquired at two different times $t_{1}$ and $t_{2}$ is defined as follows [23,24]:

(7) $$\gamma_{t_{1}t_{2}}=\frac{s_{t_{1}}\cdot s_{t_{2}}}{\sqrt{\langle {\left|s_{t_{1}}\right|}^{2}\rangle \cdot \langle {\left|s_{t_{2}}\right|}^{2}\rangle }}$$

where 〈⋅〉 represents the average of the sample. Traditional interference image processing is generally performed in a sliding rectangular window, and the coherence is calculated by combining the imaging results obtained at different times in the same monitoring area. The commonly used window size is generally an odd number. If the rectangular window is set too small, then the interference pattern will be severely affected by noise. If the rectangular window is set too large, then the coherence resolution will be reduced. Typical window sizes are 7, 9, and 11. It should be noted that this article only involved obtaining coherence through the HRRP. Therefore, this article used a 9 × 1 one-dimensional window.

## 3. Three-Dimensional Imaging Based on Slope Smoothness Constraints

Ground-based real-aperture radar scans the slope monitoring area at a constant speed according to the set parameters and obtains the target distance, horizontal angle, and elevation angle to achieve a 3D resolution. Each scan position corresponds to a single pixel on the radar image. Taking into account the actual application requirements of slope monitoring, we only needed to pay attention to the stable main scattering targets at each scanning position of the slope. Due to the influence of manmade interference (such as vehicles and mechanical equipment) and random noise in the slope monitoring environment, there might have been several scattering targets along the electromagnetic wave propagation path at each scanning position, and the corresponding HRRP might have several peak points within the selected range gate.

**Figure 3:**
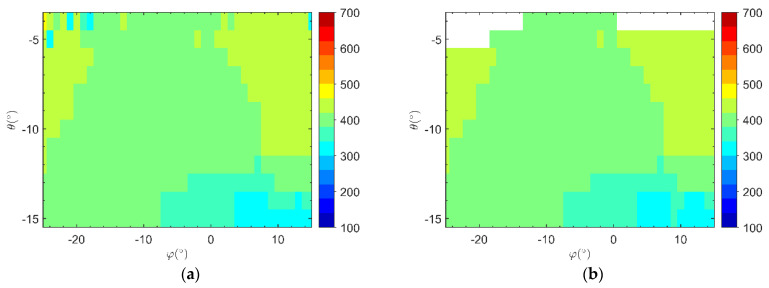
(**a**) Target slant range analysis based on amplitude threshold; (**b**) Target slant range analysis based on a coherence threshold of 0.7 and amplitude threshold.

The normalized amplitude and coherence provided foundational data for 3D slope imaging, which can serve as a basis for decision-making when acquiring 3D slope images. By studying the SSR-related user manual from Ground Probe Company, we can summarize that real-aperture radar systems usually use the amplitude threshold method for 3D imaging. However, as shown in Figure 3a,b, if the 3D images are processed only based on the peak-point amplitude threshold or the amplitude and coherence thresholds, then there may be an abnormal situation in which the imaging target deviates greatly from the spatial position of the slope. In this case, the efficiency of slope deformation monitoring and analysis will reduce.

### 3.1. Smoothness Constraint Principle

The 3D image of the slope should clearly show the contours of the monitored area. Because the spatial position of the adjacent radar monitoring spot area in the slope should conform to the approximate smooth and continuous distribution of the slope surface, we proposed a 3D imaging method based on smoothness constraints. For range function R(θ,φ), the slope gradient at coordinate (θ,φ) can be defined as a 2D vector:

(8) $$\nabla f\equiv grad\left(f\right)=\left[\frac{\partial R\left(\theta ,\varphi \right)}{\partial \theta },\frac{\partial R\left(\theta ,\varphi \right)}{\partial \varphi }\right]$$

This vector indicates the direction of the maximum rate of change of R at position (θ,φ). The amplitude (length) of vector ∇f is the direction change rate of the gradient vector at (θ,φ), which can be expressed as follows [25]:

(9) $$M\left(\theta ,\varphi \right)=\mathrm{mag}\left(\nabla f\right)\approx \left|\frac{\partial R\left(\theta ,\varphi \right)}{\partial \theta }\right|+\left|\frac{\partial R\left(\theta ,\varphi \right)}{\partial \varphi }\right|$$

The roughness of the surface function can be defined as the Frobenius norm of its Hessian matrix [26], as follows:

(10) $$r={\left||\nabla^{2}R\left(\theta ,\varphi \right)|\right|}_{2}^{2}=\frac{\partial^{2}R\left(\theta ,\varphi \right)}{\partial \theta^{2}}+2\cdot \frac{\partial^{2}R\left(\theta ,\varphi \right)}{\partial \theta \partial \varphi }+\frac{\partial^{2}R\left(\theta ,\varphi \right)}{\partial \varphi^{2}}$$

When *R* is a discrete function, the partial derivative operators in Equations (8)–(10) can be approximated by different operators.

In this paper, the slope gradient threshold and surface roughness were used as the evaluation indicators of the slope smoothness, and the final imaging targets in the monitoring area of $\left(\theta_{m},\varphi_{n}\right)$ were then determined among the candidate targets:

$$R_{i_{m,n}}\left(\theta_{m},\varphi_{n}\right),i_{m,n}\in I_{m,n}.$$

**Figure 4:**
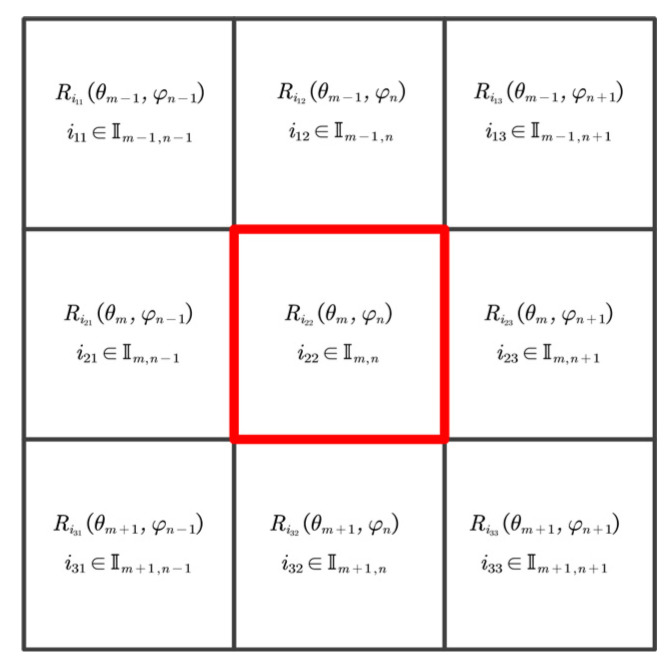
Roughness calculation window.

As shown in Figure 4, a 3 × 3 pixel window was established with the imaging target area as the center. Any target extracted from each pixel unit was used to construct a curved surface, and its roughness was calculated. All candidate targets in the area that met the gradient threshold were traversed. The candidate targets with the best smoothness were regarded as the imaging targets for the area; that is, the goal was to find the function $f\left(m,n\right)=R_{i_{m,n}}\left(\theta_{m},\varphi_{n}\right)$ that minimized the following equation under the premise that $M\left(\theta_{m},\varphi_{n}\right)$ was less than the gradient threshold $\varepsilon_{3}$:

(11) $${\left||\nabla^{2}f\left(m,n\right)|\right|}_{2}^{2}=\overset{M-1}{\underset{m=2}{\sum }}\overset{N-1}{\underset{n=2}{\sum }}\begin{array}{c} \Delta_{mm}^{2}+ \end{array}2\Delta_{mn}^{2}+\Delta_{nn}^{2}$$

where,

$$\Delta_{mm}^{2}={\left||f\left(m+1,n\right)+f\left(m-1,n\right)-2\cdot f\left(m,n\right)|\right|}^{2},$$

$$\Delta_{nn}^{2}={\left||f\left(m,n+1\right)+f\left(m,n-1\right)-2\cdot f\left(m,n\right)|\right|}^{2},$$

$$\Delta_{mn}^{2}={\left||f\left(m+1,n+1\right)+f\left(m,n\right)-f\left(m,n+1\right)\begin{array}{c} -f\left(m+1,n\right) \end{array}|\right|}^{2}$$

**Figure 5:**
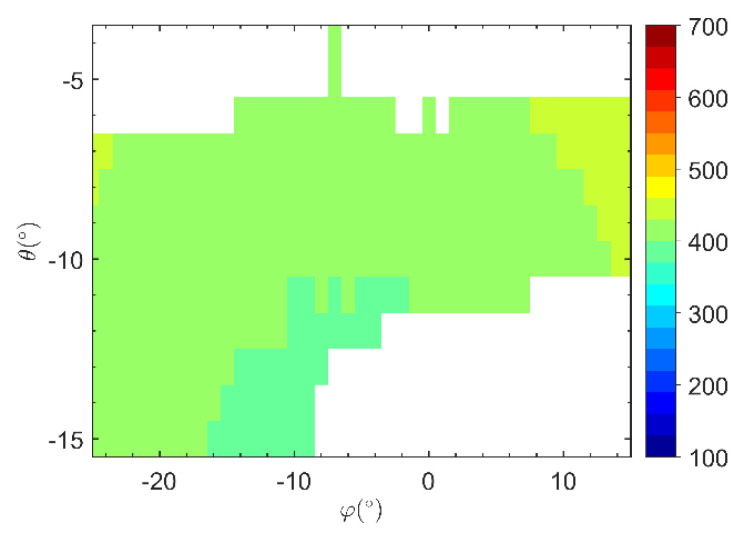
Analysis of target slant range under smoothness constraints.

In addition, the candidate targets that were closest to the imaging targets in adjacent pixels within the monitored boundary area were regarded as the imaging targets for the area:

Based on the target screening results for an amplitude threshold of $\varepsilon_{1}=0.8$ and a coherence threshold of $\varepsilon_{2}=0.9$ and according to the slope smoothness constraints, the slant range analysis results of the high-step slope imaging targets in the Heidaigou Open-Pit Coal Mine are shown in Figure 5. By comparing the two processing methods described above, it can be seen that based on the slope gradient threshold and roughness evaluation index, each imaging target can reflect a favorable slope smoothing trend, which is suitable for research on the processing of 3D images of slopes.

### 3.2. Three-Dimensional Imaging Procedures

**Figure 6:**
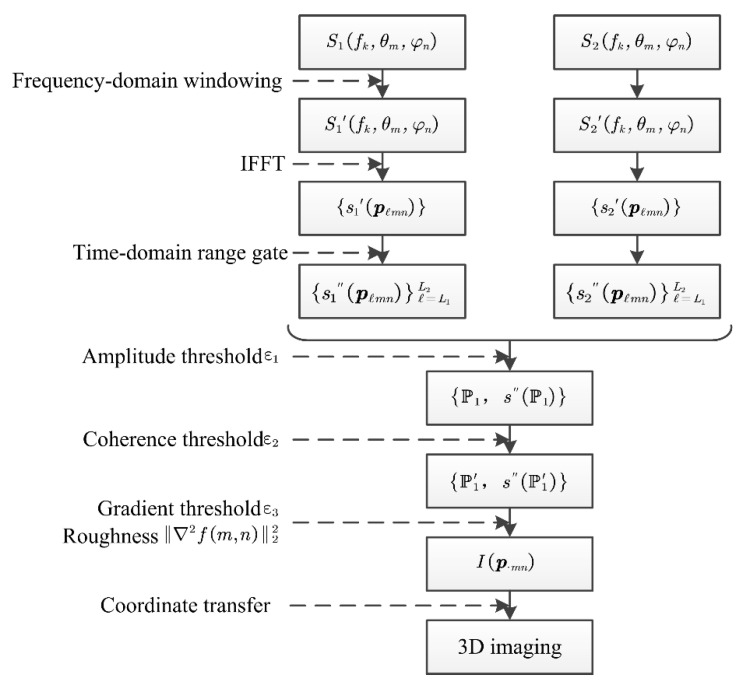
Procedure for 3D imaging of ground-based real-aperture slope radar.

It was assumed that there was a quasistatic strong scatterer target in each beam spot coverage area of the target slope, which could generate scattered echo signals with better coherence. The procedure for obtaining 3D image information $I\left(R\left(\theta_{m},\varphi_{n}\right),\theta_{m},\varphi_{n}\right)$ from the scattered echo signal $S\left(f_{k},\theta_{m},\varphi_{n}\right)$ is shown in Figure 6. The specific steps are summarized as follows:

1. Windowing processing is performed on the original echo $S\left(f_{k},\theta_{m},\varphi_{n}\right)$ in the frequency domain to reduce signal power leakage and filter out short-distance direct leakage signals and other short-distance target interference to obtain echo data $S^{\prime }\left(f_{k},\theta_{m},\varphi_{n}\right)$.
2. After up sampling $S^{\prime }\left(f_{k},\theta_{m},\varphi_{n}\right)$, the HRRP is obtained by IFFT, and the position of the peak point of the target echo amplitude in the slope can be accurately determined as follows:
  $$s^{\prime }\left(R_{\ell },\theta_{m},\varphi_{n}\right),\ell =1,2,\ldots ,L.$$
  To simplify the representation, we introduced the notation $p_{\ell mn}=\left(R_{\ell },\theta_{m},\varphi_{n}\right)$. Then the HRRP can be expressed as follows:
  $$\left\{s^{\prime }\left(p_{\ell mn}\right)\right\},\ell =1,2,\ldots ,L.$$
3. Based on the target conditions of the slope monitoring site, the time-domain range gate was set once HRRP $\left\{s^{\prime }\left(p_{\ell mn}\right)\right\}$ was obtained after IFFT:
  $${\left\{s^{\prime \prime }\left(p_{\ell mn}\right)\right\}}_{\ell =L_{1}}^{L_{2}},\ell =L_{1},L_{1}+1,\ldots ,L_{2}.$$
4. The main scatterer target in the slope was at a high level in the amplitude of the scattered echo signal of the monitoring spot. By obtaining the amplitude of ${\left\{s^{\prime \prime }\left(p_{\ell mn}\right)\right\}}_{\ell =L_{1}}^{L_{2}}$ and performing normalization and based on the amplitude threshold, the initial screening of each peak point was performed to obtain the peak point set $\left\{P_{1},s^{\prime \prime }\left(P_{1}\right)\right\}$, as follows:
  $$P_{1}=\left\{p_{\ell mn}:\left|{\left\{s^{\prime \prime }\left(p_{\ell mn}\right)\right\}}_{\ell =L_{1}}^{L_{2}}\right|>\varepsilon_{1}\right\},$$
  where $s^{\prime \prime }\left(P\right)≜\left\{s^{\prime \prime }\left(p_{\ell mn}\right);p_{\ell mn}\in P\right\}$.
5. There may be coherent speckle noise close to the target position of the main scatterer in the scattering echo at the monitoring spot. A coherence threshold was also required for secondary screening to limit the effects of coherent speckle. Using the HRRP under the same monitoring direction $\left(\theta_{m},\varphi_{n}\right)$ collected within the same period of time as the current monitoring, the coherence was calculated by the method described in Section 2.2 and denoted as $\gamma_{t_{1}t_{2}}\left(p_{\ell mn}\right)$. Then, the candidate target point set $\left\{P_{1}^{\prime },s^{\prime \prime }\left(P_{1}^{\prime }\right)\right\}$ was obtained:
  $$P_{1}^{\prime }=\gamma_{t_{1}t_{2}}\left(p_{\ell mn}\right)>\varepsilon_{2},p_{\ell mn}\in P_{1}.$$
  Note that if there was no coherent point in the spot area, $P_{1}^{\prime }$ may also be an empty set, and the corresponding direction was regarded as having no target at this time.
6. After screening by the normalized amplitude and coherence threshold, there might still be multiple candidate targets in the monitoring spot. According to the principle described in Section 3.1, the final imaging target of the area was obtained based on the slope smoothness constraint, and its corresponding slant range can be expressed as $R_{i_{m,n}}\left(\theta_{m},\varphi_{n}\right)$. Therefore, the corresponding position can be expressed as $p_{\cdot mn}=\left(R_{i_{m,n}}\left(\theta_{m},\varphi_{n}\right),\theta_{m},\varphi_{n}\right)$.
7. Repeat steps 1-6 for all $\left(\theta_{m},\varphi_{n}\right)$ in the slope monitoring area to obtain the set of all imaging targets of the main scatterers in the slope monitoring area:
  $$I\left(p_{\cdot mn}\right)={\left\{p_{\cdot mn}\right\}}_{m=1,n=1}^{M,N}$$
  This set was precisely the result of the 3D imaging in the radar polar coordinate system.
8. The radar polar coordinate system can be converted into a Cartesian coordinate system, the 3D spatial space coordinate data of the main scatterer target in the slope can be obtained, and the deformation and displacement data of the target point can be fused and displayed in the 3D spatial Cartesian coordinate system to facilitate the visual interpretation of 3D image information.

## 4. Results and Discussion

**Figure 7:**
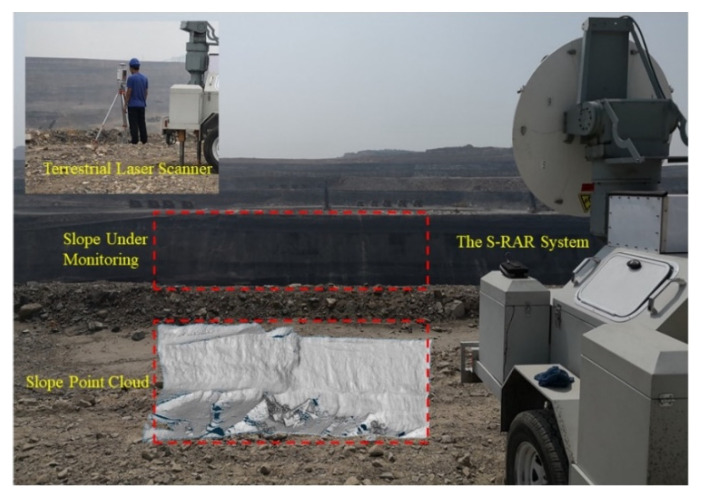
Monitoring scene optical photo and point cloud image.

To verify the effectiveness of the proposed 3D imaging method for ground-based real-aperture radar, we used the S-RAR system to conduct deformation monitoring experiments on the northern high steps of the pit in the production area of the Heidaigou Open-Pit Coal Mine and obtained slope point cloud data for the same period using a 3D laser scanner. The radar system parameter settings are listed in Table 2. The monitoring field scene and point cloud image are shown in Figure 7.

According to the abovementioned 3D imaging processing flow, Figure 2 in Section 2.2 showed the HRRP processing results in a specific monitoring direction. Figure 5 in Section 3.1 showed the distribution of the slant range of the main scatterer imaging target in the radar polar coordinate system O-Rθφ in the slope monitoring area. Note that when the elevation angle was −4° or −5°, most of the target slant range did not meet the expected range. It may be that the elevation angle setting exceeded the boundary range of the high-step slope, so it should be eliminated in subsequent image processing.

**Figure 8:**
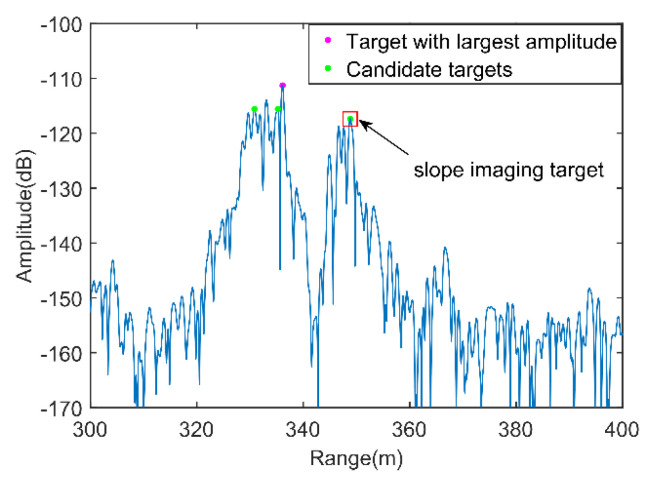
Slope imaging point analysis.

**Figure 9:**
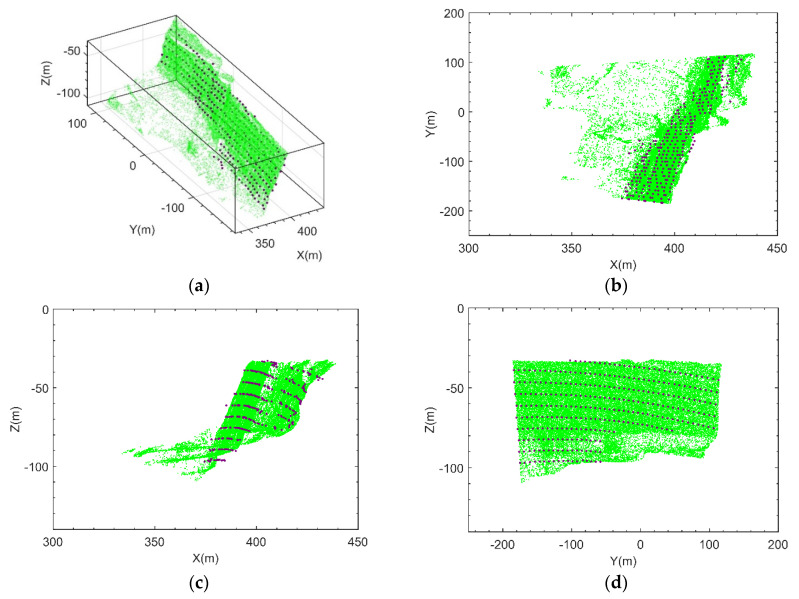
Three-dimensional imaging results comparison between the proposed method and the point cloud. (**a**) Slope 3D image comparison; (**b**) XOY projection image comparison; (**c**) XOZ projection image comparison; (**d**) YOZ projection image comparison.

Figure 8 presents slope imaging point analysis in a monitoring area, where the pink dot is the target with the largest amplitude obtained by the amplitude threshold method, the green dots are the candidate targets obtained by the method in this paper, and the dot in the red box is the slope imaging target. Figure 9a presents the three-dimensional imaging results comparison between the proposed method and the point cloud. To further analyze the imaging situation, the two images were projected in the 2D planes XOY, XOZ, and YOZ. The specific results are shown in Figure 9b–d, where O-XYZ was the spatial Cartesian coordinate system corresponding to the radar polar coordinate system.

**Figure 10:**
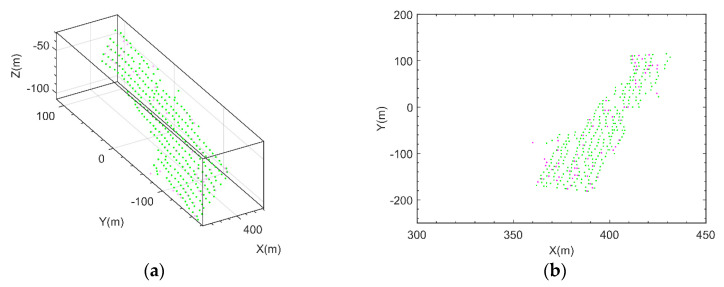

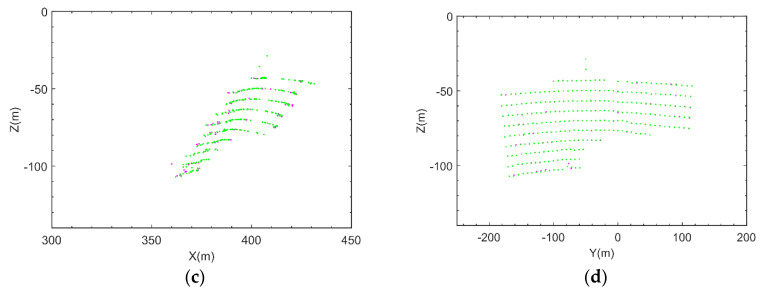
Three-dimensional imaging results comparison between the proposed method and the existing method (the green points represent the proposed method, and the pink points represent the existing method, sometimes the difference is small so that you can see only one color) (**a**) Slope 3D image comparison; (**b**) XOY projection image comparison; (**c**) XOZ projection image comparison; (**d**) YOZ projection image comparison.

In addition, the comparison with the amplitude threshold method in 3D space and 2D projection plane is shown in Figure 10, where the pink dot is the imaging targets obtained by the amplitude threshold method, the green dots are the imaging targets obtained by the smoothness constraints method proposed in this paper.

Based on the analysis of the abovementioned 2D and 3D imaging results, it is shown that the 3D imaging algorithm based on smoothness constraints can describe the spatial structure and accurately acquire the main scatterer target information in the slope space. This is conducive to target interpretation.

The performance of 3D slope imaging depends on whether the imaging target can accurately reflect the spatial distribution of slope morphology, and root mean square error (RMSE) can measure the degree of fitting of two surfaces in space. In this paper, the 3D laser scanner applied in the experiment can obtain the real slope surface almost comprehensively and accurately. On this basis, the radar image and point cloud image were resampled to a 2D projection plane using kriging interpolation to calculate the RMSE so as to evaluate the degree of matching between the radar slope 3D imaging results and the actual point cloud slope:

(12) $$RMSE=\sqrt{\frac{\sum_{\left(i,j\right)\in B}{\left[z_{rd}\left(x_{i},y_{j}\right)-z_{pc}\left(x_{i},y_{j}\right)\right]}^{2}}{n}}$$

where B is the selected interpolation area in the 2D projection plane, and $z_{rd}\left(x_{i},y_{j}\right)$ and $z_{pc}\left(x_{i},y_{j}\right)$ are the surface functions obtained by interpolation. After calculation, the root mean square error of the radar 3D image in the XOY plane was 1.0534 m. Considering that the average distance of the target in the radar image was approximately 7.7569 m and the area irradiated by the radar spot was approximately 59.8119 m^2^, the imaging error is within a reasonable range. Errors caused by issues, such as fitting the origin of the radar coordinates, servo turntable spatial positioning, and the slope inclination angle, can be analyzed and eliminated in the later stage.

**Figure 11:**
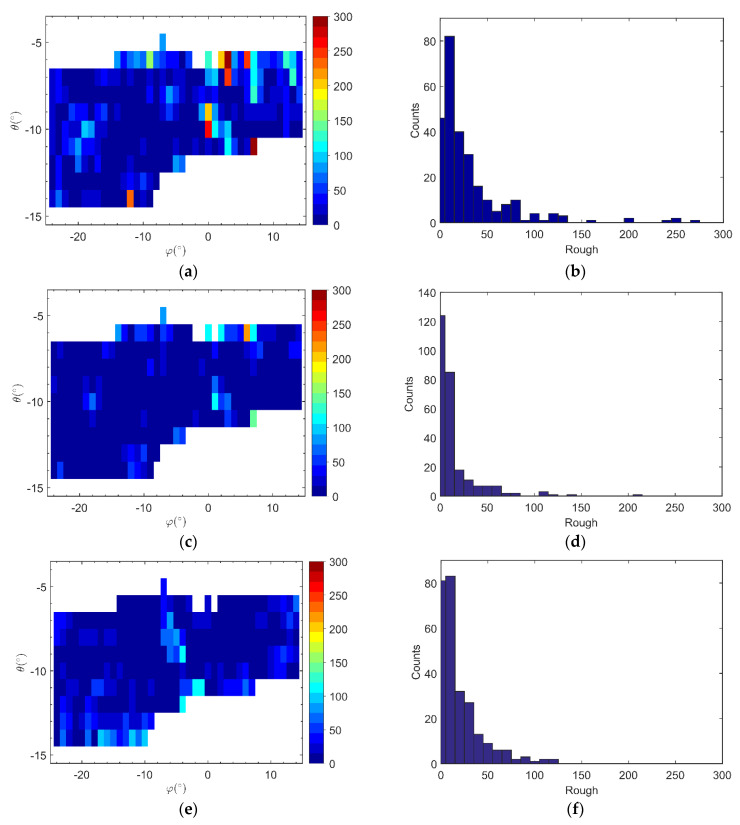
(**a**) 3D image based on existing method roughness calculation; (**b**) Roughness histogram statistics based on the existing method; (**c**) 3D image based on the proposed method roughness calculation; (**d**) Roughness histogram statistics based on the proposed method; (**e**) Point cloud image roughness calculation; (**f**) Roughness histogram statistics based on point cloud image.

In addition, surface roughness is both a basis for slope smoothness and a measure of 3D imaging performance. It can reflect the height drop between adjacent targets in the same dimension. The roughness calculation results and histogram statistics based on the existing method, smoothness constraints method, and point cloud image at the same monitoring position are shown in Figure 11. We can see from Figure 11d, the 3D imaging roughness of the slope was basically kept within 150, i.e., the distance between imaging targets was less than 6.12 m in the same dimension, which was in line with the spatial distribution of the slope area in the experimental scene.

## 5. Conclusions

Three-dimensional imaging of slopes can visually and effectively identify the location of points with a potential risk of landslides. This is of great significance for engineering safety assurance and slope disaster warning and prediction. By analyzing radar echo signal models, this paper presented a 3D ground-based real-aperture radar method for slope imaging based on smoothness constraints. Amplitude and coherence thresholds were used to eliminate some interfering targets in the HRRP. Then, according to the slope gradient and roughness analysis, strongly scattered targets in the slope space were selected for 3D imaging.

The independently developed S-RAR system was used in combination with a 3D laser scanner to carry out 3D imaging experiments on high-step slopes of mines. By comparing the radar imaging targets with the point cloud image in the 2D plane during the same time period, the effectiveness and practicability of the proposed method were verified. We also used RMSE function and roughness as two measures to analyze different imaging results. It shows that the slope imaging based on the smoothness constraints method is more suitable for actual three-dimensional slope imaging.

## Figures and Tables

**Table 1:** Kaiser window parameter analysis.

β	Peak Sidelobe Ratio/dB	Widening Factor
0	−13.26	1.00
1	−14.66	1.02
2	−18.44	1.13
2.5	−20.95	1.18
3	−23.75	1.23
3.5	−26.77	1.29
4	−29.96	1.35

**Table 2:** S-RAR parameter setting.

Carrier Frequency/GHz	Signal Bandwidth/MHz	Stepping Accuracy/°	Horizontal Range/°	Elevation Range/°
14.25	500	1	−25~15	−15~−4

## Data Availability

Not applicable.
